# Contextual assessment of perceived health system alignment with National Standards for Cancer Survivorship Care in the American Southeast

**DOI:** 10.1007/s11764-026-02123-0

**Published:** 2026-09-15

**Authors:** Shirley M. Bluethmann, Stephanie Bunch, Chandylen Nightingale, Alana R. Willis, Beth York, Beth Fisher, Sarah A. Birken, Rachel Zimmer, Carla Strom, Ashley Strahley, Sarah Zakrzewski, Stephanie J. Sohl

**Affiliations:** 1 Department of Social Sciences and Health Policy, Division of Public Health Sciences, Wake Forest University School of Medicine, Winston-Salem, USA; 2 Atrium Health Wake Forest Baptist Comprehensive Cancer Center, Winston-Salem, USA; 3 Section on Gerontology and Geriatric Medicine, Wake Forest University School of Medicine, Winston-Salem, USA; 4 Vanderbilt-Ingram Cancer Center, Nashville, USA; 5 Department of Implementation Science, Division of Public Health Sciences, Wake Forest University School of Medicine, Winston-Salem, USA

**Keywords:** Cancer survivors, Supportive care, Delivery of health care, Participatory research

## Abstract

**Purpose:**

We assessed perceived alignment of a large health system in the American Southeast with the National Standards for Cancer Survivorship Care (National Standards).

**Methods:**

We engaged key partners and distributed an electronic survey to oncology practices within the system. In parallel, in four representative clinics we collected qualitative data including four clinic observations and 12 semi structured interviews informed by the Consolidated Framework for Implementation Research (CFIR).

**Results:**

Of the 51 informants at practices who received the survey, 16 consented, and 10 completed at least one item. On average, informants reported meeting 55% of the survivorship care National Standards at least some of the time. Facilitators and barriers for National Standards implementation were identified in these CFIR Domains: Characteristics of the Individuals (e.g., believed to be beneficial for improving and standardizing care), Outer Setting (e.g., need to be linked to an incentive), Inner Setting (e.g., need for more provider training on what is available), Intervention Characteristics (e.g., difficult to understand), and Process (e.g., important decision-makers).

**Conclusions:**

While most informants reported meeting at least half of the National Standards, there was variability in how consistently National Standards were perceived as applied across practices. Further, there was likely bias, with more favorable responses from those who chose to complete the survey than those who did not.

**Implications for Cancer Survivors:**

These data demonstrate a systematic approach for assessing alignment with National Standards and identifying opportunities for improving survivorship care. A model that combines standardized survivorship services with flexibility to meet local population needs may help reduce gaps in care, promote equitable access to supportive resources, and enhance outcomes for cancer survivors across diverse settings.

**Supplementary Information:**

The online version contains supplementary material available at https://doi.org/10.1007/s11764-026-02123-0.

## Introduction

The National Cancer Institute defines a cancer survivor as any individual from the point of diagnosis through the balance of life [1]. There are currently more than 18 million cancer survivors in the United States [2]. With advances in diagnostic and treatment protocols and the changing demographics fueled by an aging population (sometimes called the “silver tsunami”) [3], this number is expected to grow. Individuals with cancer have distinct survivorship needs, including physical and psychological symptoms throughout the cancer continuum [4]. These considerations may include the risk for recurrence, new cancers, and changing social needs [4]. As a result, most survivors require a long-term period of follow-up care. Despite this identified need for continuing care, there is no consensus on the best ways to operationalize follow-up care, and the quality of survivorship care has been viewed as suboptimal in the United States.

Although there have been several sets of guidelines created to direct quality survivorship care [5–10], overall, survivorship care remains fragmented, leaving many survivors with persistent symptoms, unmet needs, and inconsistent access to coordinated services across settings [5, 8]. In response, the National Cancer Institute developed the National Standards for Cancer Survivorship Care (“National Standards”) to guide health systems on essential policies, processes, and quality evaluation, providing a consensus blueprint to address these shortcomings and improve outcomes [11]. The goal of the “National Standards” initiative was to build upon existing efforts (e.g., Commission on Cancer accreditation requirements) to develop standards for survivorship care that can be utilized by all healthcare systems to assess the quality of existing survivorship care and guide the development of new programs and services to better support cancer survivors.

We explored congruence of current practices across a healthcare system in the American Southeast with the National Standards. The objectives of the project were to: 1) identify the extent (quantitatively) to which survivorship care programs in a multidimensional health care system perceived aligning with the National Standards; and to 2) qualitatively contextualize current cancer survivorship care by identifying barriers, gap areas, and facilitators to inform future adaptation and implementation of care aligned with National Standards in a manner that is tailored to our healthcare system.

## Methods

We employed a qualitatively dominant convergent mixed‑methods design to assess alignment of current practices across a healthcare system in the American Southeast (i.e., catchment area covering urban and rural areas of North and South Carolinas, as well as parts of Georgia and Virgina) with the National Standards. Quantitative data were collected through an electronic survey distributed to oncology practices within the system to provide system‑level context for the qualitative data collected in four representative practices (two large main practices and two smaller regional practices). Qualitative data included four clinic observations and 12 semi‑structured interviews, guided by the first parts of a co-design process at Atrium Health Wake Forest Baptist Comprehensive Cancer Center (AHWFBCCC), to capture in‑depth insights into current survivorship care and factors influencing its potential implementation of additional practices to further align with the National Standards. The survey findings were integrated with qualitative themes during analysis, enabling a convergent mixed‑methods approach in which the qualitative data served as the primary source and the quantitative data played a complementary, supportive role to create a comprehensive contextual understanding of the extent to which current practices were perceived to align with the National Standards and factors that could influence future alignment. A Survivorship Insights Council, which consisted of project champions, community outreach and engagement leaders, supportive oncology and survivorship researchers, healthcare providers, and a cancer patient advocate, was consulted with at different points in the study to engage those throughout the project who had interests or involvement in survivorship care. All study procedures were approved by the Wake Forest University School of Medicine’s Institutional Review Board (IRB; study number IRB00118313).

### Quantitative: key informant survey

We adapted the Organizational Assessment Tool from the NCI National Standards for Cancer Survivorship Care Toolkit for the survey with refinements and additional clarifying questions derived from discussions with the key partners in our study convened Survivorship Insights Council. The survivorship toolkit created by the developers of the National Standards provides resources that support health systems using the survivorship standards to develop or enhance their survivorship programs. Aligned with the National Standards Toolkit [13], the key informant survey included 10 questions regarding Health System Policy (e.g., organizational policies pertaining to establishment, existence, and structure to provide survivorship care), 10 questions regarding Health System Processes (e.g., organizational processes to delivery survivorship care) and 10 questions regarding Health System Evaluation (e.g., organizational data collection of the effects from survivorship care). We integrated feedback from the Survivorship Insights Council by adding instructions, parentheses, response options, and follow-up questions. Further, consistent with survey design standards [12], we separated questions about survivors both (1) being assessed and (2) being provided with resources and/or referrals.

The additional instructions and parentheses served to facilitate understanding of terms as used in the specific context. For example, in the key definitions, we specified in parentheses that survivorship care was “including supportive oncology services offered from the time of diagnosis.” The additional response options and follow-up questions aimed to provide more nuanced data. For example, having two *yes* options for items in the Health Systems Processes section (Yes, routinely; Yes, sometimes [e.g., varies by clinician or department]) enabled collapsing yes/no data as collected in the toolkit and allowed us to differentiate any inconsistent processes within a practice. Similarly, a second *no* response option was further added for two items within Health System Processes that asked about assessing survivors at multiple points (No, only assessed once; No, not assessed at all). Follow-up questions on topics relevant to operational leaders were also added, such as clarifying how practical and social effects of cancer were assessed and referral processes for caregivers to onsite services to address their unmet needs. Further, it was of interest to our partners to collect more detailed data on which referrals were available to cancer survivors with a list of specific services. Data on follow-up questions are not reported and will inform local decision-making.

We distributed the resulting electronic survey via REDCap to 51 informants representing sites (i.e., oncology practices) within the system identified by two oncology service line leaders that included clinical champions and/or clinical team leaders. We also compiled existing data (including the cancer center registry data) to further characterize patients seen at each of the practices (e.g., number of new cancer patients, race, ethnicity, rural location, cancer types, age). The survey was sent to informants representing oncology practices that comprise the healthcare system located in North and South Carolina. This overall group of practices included four focus practices: two main oncology practices and two regional oncology practices (see Table 1).

**Table 1:** Oncology patient demographics by oncology practice, Cancer Registry Data, 2023

	All Practices^a^ (*n*=10 Clinics)	Main Campus A	A – Regional Location	Main Campus B	B – Regional Location
New patients (n)	17,268 (100.0%)	4,500 (26.1%)	217 (1.3%)	6,459 (37.4%)	1,081 (6.3%)
Location					
Rural	2,919 (16.9%)	1,158 (25.7%)	208 (95.9%)	825 (12.8%)	28 (2.6%)
Urban	14,173 (82.1%)	3,334 (74.1%)	9 (4.1%)	5,557 (86.0%)	1,023 (94.6%)
Sex					
Male	8,097 (46.9%)	2,412 (53.6%)	110 (50.7%)	3,008 (46.6%)	445 (41.2%)
Female	9,168 (53.1%)	2,087 (46.4%)	107 (49.3%)	3,450 (53.4%)	635 (58.7%)
Race					
White	13,322 (77.1%)	3,763 (83.6%)	207 (95.4%)	4,790 (74.2%)	636 (58.8%)
Black	3,291 (19.1%)	592 (13.2%)	8 (3.7%)	1,428 (22.1%)	394 (36.4%)
Asian	366 (2.1%)	41 (0.9%)	0 (0.0%)	151 (2.3%)	31 (2.9%)
American Indian/Alaskan Native	69 (0.4%)	21 (0.5%)	0 (0.0%)	20 (0.3%)	6 (0.6%)
Native Hawaiian/Pacific Islander	12 (0.1%)	5 (0.1%)	0 (0.0%)	4 (0.1%)	1 (0.1%)
Other	106 (0.6%)	59 (1.3%)	1 (0.5%)	21 (0.3%)	4 (0.4%)
Ethnicity					
Hispanic	601 (3.5%)	131 (2.9%)	6 (2.8%)	252 (3.9%)	56 (5.2%)
Age					
0–17	159 (0.9%)	59 (1.3%)	0 (0.0%)	96 (1.5%)	1 (0.1%)
18–64	7,731 (44.8%)	1,904 (42.3%)	87 (40.1%)	3,083 (47.7%)	526 (48.7%)
65+	9,378 (54.3%)	2,537 (56.4%)	130 (59.9%)	3,280 (50.8%)	554 (51.2%)

^a^One regional clinic that completed a survey is missing from demographic analysis due to lack of cancer registry data.

### Qualitative: clinic observation and semi-structured interviews

To complete the context assessment, we adapted Context-Driven Co-Design templates developed based on the Consolidated Framework for Implementation Research (CFIR) [13, 14] including an interview guide (please see Supplementary Material) and observation tool [15]. We purposively selected four focus practices for the clinic observations to represent a range of resources and patient characteristics (regarding race, ethnicity, and rural/urban location). We rapidly assessed each practice’s current survivorship care context to identify factors perceived to support or hinder alignment with the National Standards and explored strategies that may enable alignment in next steps. We included the following initial components of the Context-Driven Co-Design approach: First, to understand current survivorship care and other factors influencing survivorship care within the four selected practices, we conducted clinical observations and semi-structured interviews with providers, staff, and clinical leader(s) who currently or may in the future engage with survivorship care.

Clinic observations varied by the four representative practice locations and were conducted by a research associate who directed this study and facilitated other institutional cancer survivorship efforts (SB). The clinic observations were rooted in focused clinic ethnography principles [16–18] to understand the context of the users (i.e., clinical staff) and the environment to identify potential barriers and opportunities. Ethnographic research can identify contextual barriers to health care improvement and has been identified as a research method that can show what happens routinely in health care [19]. Interviews with key invested partners from the four focus practices were designed to provide deeper insights into survey and clinic observation results. Field note observations were documented and reported to produce an ethnographic description of each of the four focus practices through user characteristics, user tasks, and the physical environment [20].

The Wake Forest Qualitative and Patient Reported Outcomes (Q-PRO) Shared Resource and the study director (SB) conducted interviews with providers and administrators at each practice to gain qualitative data regarding survivorship care practices and identify areas of strengths and opportunities for each practice. Interviews were conducted in person or via video conference. Responses to open-ended questions and probes were audio-recorded and transcribed verbatim, excluding identifying information. Interviews lasted an average of 27.5 minutes each, and participants received gift cards with a value of $20 for their time.

Next, we engaged relevant partners from the focus practices to review initial findings and prioritize what was shared with the larger groups. Collaboration with practice-specific partners, including health care administrators, is essential to translating the National Standards into action [21]. We then sought input from the AHWFBCCC’s Community Advisory Board (a standing group of volunteers including patients, caregivers, cancer advocates, local healthcare providers, and faith leaders, that serve as a link between the cancer center and the communities) and reconvened the Survivorship Insights Council to discuss the findings and priorities for implementation across practices. Finally, we summarized and shared findings that would inform future alignment with the National Standards.

## Data analyses

Descriptive statistics, including frequencies and percentages, were used to summarize survey responses across the three domains assessed: Health System Policy, Health System Processes, and Health System Evaluation, as well as practice-level demographic characteristics. For process-related items, responses of “Yes, routinely” and “Yes, sometimes” were combined to indicate any level of implementation. Rurality was classified using Rural–Urban Commuting Area (RUCA) codes, with codes 1–3 designated as urban and codes 4–10 as rural [19]. Oncology practices were included in analyses if they responded to at least one survey item. One regional practice was excluded from demographic analyses due to missing cancer registry data. Descriptive data from the individual National Standard survey items within each domain were selectively highlighted to support qualitative findings.

Interviews and observation notes were analyzed using a rapid analysis approach [22]. A Q-PRO researcher (AS) constructed a matrix in Microsoft Excel, with domains informed by the Consolidated Framework for Implementation Research (CFIR) [13] and the study-specific questions. The researcher then reviewed the interview transcripts and populated the matrix with raw data (e.g., direct quotations) corresponding to each participant. The researcher also reviewed observation notes for each practice and added these to the matrix, mapped to the CFIR construct they addressed. Then the researcher iteratively synthesized the data, first summarizing responses by individual participants, and then across partners at each practice location to identify key findings by practice.

After independent analysis of the surveys and interviews, a joint display was constructed to facilitate comparing facilitators and barriers related to implementation of the National Standards (see Table 4). Quotations were drawn from the qualitative data that aligned with specific individual standards to illustrate what facilitated or could be a barrier to meeting that standard.

## Results

### Key informant survey

Out of the 51 informants contacted, 16 consented, and 10 completed at least one survey item. Informants who completed the survey included various roles within each practice (e.g., administrator, nurse, physician, and survivorship care lead). The unique practices with completed surveys had an average of new cancer patients seen annually ranging from 217 to 6,459 and identify as 16.9% rural and 82.1% urban with an almost equal distribution of sex assigned at birth (46.9% male, 53.1% female) in 2023. The differences of rurality also varied by main oncology practice (12.8%−25.7%) and regional oncology practices (2.6%−95.9%). Patients seen within these clinics identified as 3.5% (2.8%−5.2%) Hispanic on average and 19.1% Black (3.4%−36.4%).

Informants (*n*=10) indicated meeting 55.4% of the National Standards at least some of the time, with most practices meeting areas within Health System Processes domain (see Table 2). Within Healthy System Policy, 88% (*n*=7) of key informants reported not having a policy that includes a collection of longitudinal data on survivors’ experience of survivorship care and patient reported outcomes. Most key informants -- 70% (*n*=7) -- reported having a policy that includes “establishment or existence of a survivorship program either on-site, through telehealth, or by referral.” Adherence to the National Standards for Health System Processes was high, with 91% of respondents reporting that standards were met at least some of the time. Only one respondent indicated that a single survey item was never met: access to appropriate specialty care services for managing potential late effects (e.g., cardiovascular issues) via on-site care, telehealth, or referral.

**Table 2:** “Yes” Survey Responses by All Key Informants and by Practice Location

“Yes” Survey Responses (% Yes/Items Completed)^b^	All Survey Respondents	Main Campus A	Regional Location A	Main Campus B	Regional Location B
Policy	36/85 (42.4%)	0/10 (0.0%)	0/10 (0.0%)	7/10 (70.0%)	0/4 (0.0%)
Processes	69/76 (90.8%)	10/10 (100.0%)	10/10 (100.0%)	10/10 (100.0%)	-
Evaluation	23/70 (32.9%)	3/10 (30.0%)	0/10 (0.0%)	5/10 (50.0%)	-
Total	128/231 (55.4%)	13/30 (43.3%)	10/30 (30.0%)	22/30 (73.3%)	0/4 (0.0%)

^b^For Process-related items, the ‘Yes’ category combines the response options ‘Yes, routinely’ and ‘Yes, sometimes’.

Notably, most informants reported not meeting Health System Evaluation survey items. No informant reported meeting the National Standard regarding having a process to collect data on survivors’ return to previous participation in work or other productive activities of living. Additionally, 86% of informants reported not having a process to collect data on the number of health professionals trained to provide survivorship care at their practice.

### Clinic observation and semi-structured interviews

Overall, qualitative data showed that survivorship services varied across practices. Access to survivorship care and supportive services was more extensive at main campus locations compared to regional practices. At regional practices, survivorship visits were limited to once per week or less, and related supportive services were offered in person on a limited basis or via telehealth.

Time spent observing activities within each clinic varied from approximately four hours to six hours. This involved informal discussions with partners, and participation in staff huddles, as well as observation of clinical and administrative duties and patient visits. Four patient visits were observed varying from approximately 30 minutes to one hour. Clinic observations included staff from various roles (e.g., nurse managers, physicians/APPs, clinical staff).

Interviews were conducted with 12 invested partners across the four focus practice locations (two main campuses with two regional locations as described in Table 1): Main Campus A (*n*=3), Regional Site A (*n*=3), Main Campus B (*n*=5), and Regional Site B (*n*=1). The partners interviewed represented various roles including administrator (nurse manager, director, etc.), social worker, program manager, and provider/advanced practice provider (APP). Most partners had little to no familiarity with the National Standards, outside of the context of the project. Partners shared mostly positive perceptions about implementation of the National Standards to improve patient care.

Key facilitators and barriers for National Standards implementation within each practice were identified across CFIR domains: Intervention Characteristics (e.g., challenges with comprehension), Outer Setting (e.g., requirement for linkage to incentives), Inner Setting (e.g., need for more provider/staff training regarding available resources), Characteristics of Individuals (e.g., perceived benefits in improving and standardizing care), and Process (e.g., involvement of key decision-makers) (see Table 3). Additionally, opportunities to enhance survivorship care across the health care system were identified.

**Table 3:** CFIR constructs and definitions used for analysis

CFIR Domain	CFIR Construct	Study-Specific Definition
Intervention Characteristics	Adaptability	The degree to which the National Standards can be adapted, tailored, refined, or reinvented to meet local needs
Outer Setting	Patient Needs & Resources	The extent to which patient needs, as well as barriers and facilitators to meet those needs are accurately known and prioritized by the organization
	External Policies & Incentives*	External strategies to spread interventions including policy and regulations (governmental or other central entity), external mandates, recommendations and guidelines, pay-for-performance, collaboratives, and public or benchmark reporting
Inner Setting	Implementation Climate*	The absorptive capacity for change, shared receptivity of involved individuals to the National Standards, and the extent to which use of the National Standards will be rewarded, supported, and expected within their organization
Characteristics of Individuals	Knowledge & Beliefs	Individuals’ attitudes toward and value placed on the intervention as well as familiarity with facts, truths, and principles related to the intervention
	Self-efficacy	Individual belief in their own capabilities to execute courses of action to achieve implementation goals
Process	Engaging (Key Partners)	Attracting and involving appropriate individuals in the implementation and use of the intervention through a combined strategy of social marketing, education, role modeling, training, and other similar activities

^*^Emerging CFIR theme in analyses (not explicitly asked in interview guide)

## CFIR domain: intervention characteristics

Findings identified within Intervention Characteristics (when asked about Adaptability) highlight challenges with comprehension, specificity, and accommodations for health-related social needs regarding the National Standards. Partners perceived barriers to implementation of the National Standards, describing the National Standards as not being specific enough, and thought the language of health system policies and health system processes would be difficult for people to understand, and suggested dropping policy and combining it with process. Additionally, a partner noted that the National Standards are too broad, which would hinder implementation.

[…] they're then a nice combination of vague, which helps you be able to make it fit, but again, not necessarily specific enough. And the verbiage of duplicating between policy and process is incredibly difficult for people to understand why and how that would be different. […] it's easier to operationalize something that they're saying you need a policy for X, you need a program for Y, that kind of thing. I would almost say get rid of the whole column about policy and where they may have something that isn't protocolized in the second column there, then pay attention to how that would then operationalize. (P06)

One remarked that the National Standards do not accommodate health-related social needs assessment outside of a cancer context, and that the National Standards are necessarily broad and may not fit every patient’s specific situation:

The patient goes to their primary care physician, they might be assessed for social determinants. It might not be social effects of cancer and it’s treatment, like risks and health-related needs, but they’re going to be asked if they’re safe at home. They’re probably going to be asked about food insecurity […] It’s hard for me to look at these and see that the patient exists in an oncology vacuum because they don’t […] (P05)

## CFIR domain: outer setting

Partners identified a lack of survivorship care offerings at regional practices, as well as patient knowledge gaps to survivorship care visits as barriers to implementing the National Standards. These health-related social needs were identified as contributing to patients missed or delayed appointments. Additionally, one regional clinic noted that they do not use specific measures to assess these needs.

### Patient needs and resources

Partners shared that patients face challenges such as transportation challenges, food insecurity, financial support, inadequate housing, and limited health literacy, representing both material and non-material health related social needs, within the clinic observations Additionally, partners felt that their clinic was doing a good job addressing survivors’ needs despite the absence of a designated survivorship care program, while also noting there are additional services they could offer if such a program were established. Furthermore, partners remarked that patients from regional or remote locations are often reluctant to travel to the main campus or other locations for survivorship care or supportive services. Virtual care at home is not always feasible due to limited internet connectivity in some rural areas. As a result, regional sites recommended providing survivorship care locally, either through in-person visits or via virtual visits conducted onsite:

A lot of our patients […] don't have the Internet access where they can just do a virtual visit on their phone because they lack that Internet access or the cellular access because of being on top of the mountain. [...] If we have to send our patients to the main campus, they're like, we don't want to go to, we don't want to go to main campus. We don't want to deal with that parking deck. (P03)

Partners felt that patients are not aware of the purpose of survivorship care and communicated with the need to educate patients about what survivorship care includes, to facilitate their buy-in and interest in survivorship care. A regional partner remarked that survivors may not perceive survivorship care as a priority, so there may be a need for additional patient education in order to increase uptake of survivorship care.

I think patient education goes a long way when we're talking to patients about the need and the necessity of tracking their cancer even after they finish chemotherapy. It doesn't just go away. The journey doesn't stop that day. I think that that's definitely something that would have to be explained well to those patients. Patients perceive it now as unnecessary, sometimes. You just really have to encourage those patients that this is what we're doin' to help keep you in remission, keep you cancer free. Things like that. (P01)

### External policies and incentives

Partners reported that incentives would facilitate National Standards' implementation. One partner suggested that increased attention toward survivorship care, through the National Standards, would be beneficial but would be difficult to operationalize at present because they were not linked to incentive structures or accreditation requirements.

Linking the National Standards to accreditation (e.g., American College of Surgeons Commission on Cancer [CoC])[23] or an incentive could facilitate adoption of the National Standards within oncology practices:

While it's a good idea, and the time has come for more definition in it, it's almost impossible to do with competing priorities unless it's an essential part of what we're asked to do […] we're a larger institution that has the means and resources to do it. Smaller ones really would never be able to do it unless it was part of a requirement. And again, COC [Commission on Cancer] is the way to do the requirement. (P06)

## CFIR domain: inner setting

Partners identified the need for more provider/staff training regarding available supportive oncology services as well as the need for sufficient staffing to facilitate access to these supportive services. For example, one partner explained that there were likely resources available within the organization, but that staff and providers at regional sites are not aware of what is available at other practice locations.

I don’t think there’s a lack of resources. There’s not a lack of resources. I think what it is a lack of knowledge—what resources are available […] their main focus is to get the patient in, get them treated, and get them out the door […] (P04)

It was suggested that additional staff are needed to support survivorship care and facilitate access, including more navigators, social workers, and mental health counselors. Other resource needs varied by site, but included improved tracking of survivorship care, improved health-related social needs screening and referral processes, and training on survivorship care for practice staff. Access to survivorship care and supportive oncology services was more extensive at main campus locations compared to regional practice sites. At regional sites, survivorship care may be limited once per week or less, and supportive services are offered in person on a limited basis, or via telehealth. A partner from a regional practice shared:

Quite frankly, they're getting the bare minimum when it comes to survivorship. […] If I'm seeing a breast follow up who's completed treatment, it's focused on their breast, not making sure they've had their colonoscopies, making sure they're having their lung screenings and all that stuff, because we just we don't have the time to do that. And so somebody I've done a little bit of survivorship, where I came from, but it could definitely we could definitely use a lot of work with that here. (P03)

To mitigate some of these challenges, one partner recommended an electronic medical record tool that can pull information into the visit summary for patients to read before they come in for their survivorship visit to facilitate patient understanding on the purpose of survivorship care:

[...] I think the biggest thing is communication about what the survivorship visit is and it's not just a box that's being checked off and a referral that's being placed with really no

input from the physicians, but being able to offer at the time of referral from the

physicians, being able to offer a little bit of information. I don't know if there if it would be of any benefit to make like a small Epic template that you can put in the visit summary

just to give people something to read before they come so they understand what the visit

is and how important it is. (P07)

## CFIR domain: characteristics of individuals

Regarding individuals’ knowledge, beliefs and self-efficacy, most partners had little to no familiarity with the National Standards, outside of the context of this study. Partners shared mostly positive perceptions about implementation of the National Standards as facilitators to improve and systematize patient care (see Table 4).

**Table 4:** Joint display analysis

Quantitative Findings	Example Qualitative Finding	Conclusions
Health System Policy		
• 67% of practices reported having a policy on the overview of how to stratify & refer survivors to appropriate models of care based on age, treatments, and risk factors • 50% of practices reported having an outline for the provision of information for supportive services based on survivors’ needs • 38% of practices reported having a policy description of the approach & timing of transitions in survivorship & shared care • 56% of practices reported having a policy description of multidisciplinary care including specific roles/responsibilities & workflow(s) for referrals	**Facilitator & Barrier** *“While it's a good idea, and the time has come for more definition in it, it's almost impossible to do with competing priorities unless it's an essential part of what we're asked to do […] we're a larger institution that has the means and resources to do it. Smaller ones really would never be able to do it unless it was part of a requirement. And again, COC [Commission on Cancer] is the way to do the requirement.” (P06)* **Facilitator** *“I think it's a good outline. I think it's important. I think it's important to have an established survivorship program. I think it's important to have guidelines to follow. I think it's important to be able to offer this information to patients. I think it's important to give them an idea of things that they've been through and things that we will continue to do during their care as they're healing through their cancer.”(P07)*	Practices reported mostly meeting Health System Policy standards for survivorship care. Qualitative findings suggest overall general support for the general idea of the National Standards. Also, that vague or overlapping policy language, competing priorities, limited resources, and lack of clear guidance for multidisciplinary care and transition to post treatment care contribute to inconsistent implementation, thus, highlighting the need for clearer and more actionable policies to support survivorship workflows.
Health System Process		
• 62% of practices reported yes to and 100% of practices reported at least sometimes, having a process of assessing patients at multiple points in their follow-up care for emotional and psychological effects of cancer and its treatment and provided with treatment or referrals • 50% of practices reported yes to and 100% of practices reported at least sometimes assessing patients for practical and social effects of cancer and its treatment and providing patients with resources and referrals • 50% of practices reported yes to and 100% of practices reported at least sometimes providing patients with access and referral to a survivorship program addressing the needs of survivors on-site, telehealth, or by referral • 71% of practices reported yes to and 100% of practices reported at least sometimes assessing patients for financial hardship/toxicity and concerns about insurance coverage and provided with resources as needed • 86% of practices reported yes to engaging in the care planning process, including the discussion of shared goals of care, advanced care planning, and coordination with providers and services as needed while 100% of practices reported engaging in this care planning process at least sometimes	**Facilitator** *“[...] I think the biggest thing is communication about what the survivorship visit is and it's not just a box that's being checked off and a referral that's being placed with really no input from the physicians, but being able to offer at the time of referral from the physicians, being able to offer a little bit of information. I don't know if there if it would be of any benefit to make like a small Epic template that you can put in the visit summary just to give people something to read before they come so they understand what the visit is and how important it is.”(P07)* **Barriers** *“As long as the patients don't have to travel down to—outside of their local area—because a lot of times it is transportation problems or it is financial problems and then they don't want to go out of their local community.”(P04)* *“Half the people have no idea why they’re here when we start the visit. And so, I mean, I always ask now when I go in, if they're like aware of what the purpose of the visit is, and half the people have no clue. They just know that it showed up on their schedule.”(P07)*	While all practices reported addressing survivorship needs at least sometimes, fewer practices did so routinely. The findings suggest that while survivorship care is offered, some practices may have the resources to do it and some may not, which contributes to variability in cancer-specific support. Although practices generally assessed survivor needs and engaged in care planning, limited staffing, time constraints, and variable access to survivorship programs, particularly in regional and rural settings, hindered routine integration into follow-up care. The qualitative findings highlight gaps in communication and knowledge among clinicians, staff, and patients – this emphasizes the need for routine survivorship education, increased staffing and services, and expanded, accessible survivorship resources to support comprehensive post-treatment care.
Health System Evaluation		
• 33% of practices reported having a process to collect data on: o Survivors’ patient-reported outcomes, including quality of life, and experiences of survivorship care o Survivors’ functional capacity o Survivors’ return to previous participation in paid and unpaid work/school/productive activities of living o Relevant business metrics to show return on investment of survivorship care to the healthcare system	**Facilitator & Barrier** *“It probably would give more consistency to how the clinics are being run or more guidance to the providers that are running those clinics. Maybe they are collecting data. I don't know, but I think if you're collecting data, then it tells you the work of those clinics. [...] Compared to we have a breast provider that runs a clinic, a different provider that runs GI, a different provider that runs Head and Neck. I don't know if they're all doing things consistently using the same intake processes, and it may be a little different for each disease.”(P02)*	Few practices reported evaluating survivorship care using patient-reported outcomes, which limit consistency of survivorship care. Findings indicate that limited and inconsistent data collection across clinics, which elevates the need to standardize where, how, and who collects data and how it can be accessed. The findings highlight a need for standardized methods to track survivorship visits, the services provided, and outcomes assessed to create more consistent survivorship care and shared with staff.

## CFIR domain: process

### Engaging (key partners)

The key partners identified as necessary to engage with implementation of the National Standards included clinicians (physicians, nurses, others responsible for identifying and referring patients) and those in administrative and leadership roles with decision making over hiring additional providers to deliver survivorship care. A partner interviewed also spoke to uncertainty about the structure of leadership within the enterprise, given recent changes, and not knowing whether specific individuals were in decision-making positions.

It's going to be administration. So I can tell you [other providers] and I are all going to be on the same page. These patients need supportive oncology. […] You're going to have the support from us. This administration wanting to budget—I don't know if they currently in their processes of doing the budget for the next year or not, but they need to start budgeting those FTEs to get these people hired and in place to help with your sites. (P03)

## Mixed methods integration

Findings integrated from qualitative and quantitative data are provided as a joint display (see Table 4). In both the key informant survey and interviews, partners generally found that they were meeting survivorship care standards and addressing survivors’ needs, but implementation of the National Standards was inconsistent. Barriers included unclear policy of language, limited resources and staffing, time constraints, and gaps in communication and education, particularly in regional settings, which contributed to variability in survivorship care delivery. Overall, these findings highlight the need for clearer, standardized processes to support survivorship care across practices.

## Discussion

This mixed methods study provides a comprehensive context assessment of oncology practices’ perceived alignment with recently developed National Standards for Cancer Survivorship Care across a large, multi-site healthcare system in the southeastern United States. By integrating data from a key informant survey, clinic observations, and partner interviews, our findings highlight the expected value of implementing the National Standards to improve care, existing processes that will facilitate such implementation and challenges to address to improve survivorship care delivery. Overall, practices reported meeting just over half of the National Standards at least some of the time, with strengths concentrated in the domain of Health System Processes and notable deficiencies in Health System Policy infrastructure and Health System Evaluation mechanisms as described in further detail in the following paragraphs. These findings underscore the complex interplay between structural resources, workflow organization, and evaluation practices that shape survivorship care implementation.

Practices reported meeting most Health System Policy Standards and supporting the general idea of having such National Standards. One of the most commonly met National Standards in this section (i.e., having a policy that includes establishment or existence of a survivorship program either on-site, through telehealth, or by referral) was likely motivated by already established CoC accreditation requirements [23], which include development and implementation of a survivorship program for patients treated with curative intent. The CoC requirements further delineate appointing a survivorship team and evaluating a minimum of three survivorship services that address the needs of survivors offered each year. CoC accreditation requirements were also raised in qualitative data as an example for a successful incentive to facilitate change. These findings underscore the importance of linking the National Standards to established accountability or accreditation mechanisms, such as the CoC framework, that can incentivize institutions to prioritize and sustain adoption. The National Standards aim to build upon foundational work by CoC [11]. Qualitative findings also suggest barriers to National Standards implementation that highlight the need for clearer and more actionable policies that clearly link to the Health System Processes Standards to support survivorship workflows, which may include additional staffing, infrastructure and evaluation systems

Practices reported meeting 91% of the Health System Process Standards at least some of the time. Yet, there was variability in how consistent practices met the National Standards. Key partners interviewed saw the value in applying the National Standards to facilitate standardization of enhancing care across practices, while allowing for some local tailoring (e.g., to disease, context). Another study on cancer survivorship care also found inconsistencies in provider referrals and patient awareness of relevant services [10]. Key partners also described some barriers that would need to be overcome related to gaps in communication and knowledge among clinicians, staff, and patients, emphasizing the need for more education, staffing, and accessible survivorship resources. The need for additional training of providers about cancer survivorship care (in addition to the specific resources available in each practice) has been recognized as a broader challenge across cancer centers in the United States [24]. An example of an inconsistently applied process in this study was that 50% of practices reported routinely assessing and addressing practical and social effects of cancer and its treatment. Clinic observations also revealed differences among practices in how health-related social needs were assessed and addressed [25]. Such variability in health-related social needs assessment also occurs in other clinical care settings. Further, an interviewee remarked that the National Standards do not currently consider how social needs are being assessed outside of a cancer context. Therefore, there may be opportunities to build on established processes and strengthen best practices within the broader health system to assess and address health-related social needs among individuals affected by cancer [26–29]. Evaluating how such needs contribute to missed or delayed appointments may also inform prioritization of resources.

Overall, few practices reported meeting the Health System Evaluation Standards suggesting that standards in this domain may be among the most challenging to implement. The current limited and inconsistent data collection across clinics demonstrates a need to be more strategic about what data are being systematically collected. Enhancing health system evaluation of the National Standards may require new infrastructure to be realized since the processes are not yet in place, which would take an organized system-level processes whereas other standards may be met by individual or clinic-level changes. There is an ongoing local research effort in the health system that may help establish such an infrastructure for assessing and addressing patient-reported symptoms during cancer treatment through the electronic health record and pave the way for future improvements in evaluation [30]. Other cancer centers have created data collection tools that are integrated in the electronic health record (e.g., program metrics, survivorship care plan delivery, electronic order set for referrals, smart phrases for documentation) and may inform future enhancements of current data collection methods (e.g., cancer registry [31, 32]).

## Limitations and future directions

Most informants contacted did not provide survey responses, and there was limited representation of practices among survey respondents. Therefore, there is potential response bias such that those who chose to respond to the survey may have been more engaged in survivorship care and thus the results regarding the number of National Standards reported as met were likely higher in the practices represented in the data than in those who did not complete the survey. Flexibility regarding timing for distributing the survey around competing demands, simplifying the National Standards, or further communication from leaders around the importance of completing the survey may improve future efforts. The modifications we made to the current NCI National Standards for Cancer Survivorship Care Toolkit, such as adding more nuance to the response options, was seemingly not sufficient to make the survey acceptable to key informants and further changes may be necessary prior to use in other settings such as those suggested in qualitative data related to Intervention Characteristics (e.g., be more specific, use more understandable language, consider how each National Standard is actionable [could combine policy and process], link to an incentive, include what is already being assessed in the broader healthcare context). Because the NCI definition includes individuals both active treatment and post-treatment, differences in survivorship needs and perspectives may have influenced participant responses.

Future directions may also include more objective assessments of National Standards using methods such as extracting electronic health record data. The format of clinic observations differed based on preference, which may have introduced additional variability. Yet, we chose this approach to increase engagement and reduce participant burden. The number of interviews was also limited to some practices, and additional interviews may have elicited further information. Future studies may also consider asking more explicitly about each National Standard individually, especially regarding feasibility of addressing identified barriers and suggested changes to facilitate prioritization of next steps. Yet, we still expect that the qualitative data represent the relevant topics of most importance to those interviewed and we did not attempt to complete a comprehensive assessment.

## Conclusions

In conclusion, this study highlights both the promise and challenges of implementing National Standards within a complex healthcare system. While many practices demonstrate commitment to supporting survivors, current variability in delivery, resource constraints, and lack of standardized infrastructure limit the consistency and reach of survivorship care. Addressing these gaps will require coordinated efforts across organizational levels, with particular attention to context-specific adaptation, workforce capacity, and policy alignment. This integration of quantitative and qualitative findings offers actionable insights to inform the refinement and implementation of the National Standards, with the goal of advancing equitable and high-quality care for cancer survivors.

## Supplementary Information

Below is the link to the electronic supplementary material.

ESM 1 (DOCX 43.0 KB)

## Data Availability

The datasets generated and/or analyzed during the current study are not publicly available because they contain information that could compromise participant confidentiality but are available from the corresponding author on reasonable request and with appropriate Institutional Review Board approval, where applicable.
