# Supplementary material for: Contextual assessment of perceived health system alignment with National Standards for Cancer Survivorship Care in the American Southeast
Source: J Cancer Surviv. 2026 Sep 15;20(5):1790–801. doi: 10.1007/s11764-026-02123-0 (PMC13723634; doi:10.1007/s11764-026-02123-0)
Supplement: Supplementary file 1 [file 11764_2026_2123_MOESM1_ESM.docx]

***INTRODUCTION (5 min)***

Hi, my name is _______. Thank you for agreeing to speak with us today. The purpose of this study is to assess the current infrastructure of cancer survivorship and other services, including supportive oncology, across Atrium Health Wake Forest Baptist Comprehensive Cancer Center or Atrium Health Levine Cancer practices with the ultimate goal of understanding how Atrium Health’s current cancer care aligns with the new National Standards for Cancer Survivorship Care.

Our discussion should last about 30-45 minutes. All of your responses will remain confidential within this interview. You may choose to stop the interview at any time, and there is no penalty to you for not completing the interview.

We will keep your identity confidential, making every effort to ensure that your specific responses are not attributable to you, although we do plan to report findings from this interview in aggregate along with findings from interviews with others.

Taking part in this research is not a part of your job responsibilities, and refusing will not affect your job. You will not be offered or receive any special job-related consideration if you take part in this research.

Before we begin, we would like to ask your permission to audio record our discussion. Would it be OK with you if I record our discussion? The recording will be used for training and transcription purposes. The interview will be transcribed, however, your name or any personal identifiers will not be associated with any of the notes. The audio recordings will be deleted once the project is complete. If you have any questions, you can reach out to XXX, the PI [XXemailXX].

Do you have any questions before we begin?

***INTRODUCTION (cont.)***

[Interviewer note: “probing” questions are indicated in italics.]

To start, please tell me a little bit about your role within **[PRACTICE NAME]**? To what extent are you familiar with the National Standards for Survivorship Care?

*For those that say “they* ***are*** *familiar”:*

- What are your initial thoughts about the National Standards for Survivorship Care?
  - *Probe: How have you ever heard of these new National Standards for Cancer Survivorship Care? Did anyone tell you about it and what did you think about the standards? [Leadership Engagement]*
- Would you like for us to proceed with a PowerPoint presentation that shares a high-level overview of the standards as a reminder or proceed with the interview?

*For those that say “they* ***are not*** *familiar”:*

- We will now share an overview of the National Standards for Cancer Survivorship Care to orient you [share PowerPoint].

***MAIN QUESTIONS***

| **CFIR Domain** | **CFIR Construct and Definition** | **Questions & Probes** | |
| --- | --- | --- | --- |
| Intervention Characteristics | **Adaptability**  The degree to which the National Standards can be adapted, tailored, refined, or reinvented to meet local needs | 1. Referring to the newly released National Standards for Cancer Survivorship Care, what kinds of changes or alterations do you think you would need to make so these standards will work effectively in your setting?    a. Would these changes need to vary within your practice (e.g., by clinic)? 2. What would be the process for deciding whether changes are needed so that these standards will work well in your setting? | |
|  | **Complexity**  Perceived difficulty of implementation of the National Standards for Cancer Survivorship Care, reflected by duration, scope, radicalness, disruptiveness, centrality, and intricacy and number of steps required to implement | 1. How difficult would it be to implement these new National Standards for Survivorship Care?    a. *Probe: please consider the following aspects: scope, intricacy and number of steps involved.* | |
| Outer Setting | **Patient Needs & Resources**  The extent to which patient needs, as well as barriers and facilitators to meet those needs are accurately known and prioritized by the organization | 1. How do you think the new National Standards for Cancer Survivorship Care will help identify and prioritize the needs of the survivors served by your practice? | |
| Inner Setting | **Structural Characteristics**  The location, size, age, and groups that need tailored services | 1. How will the infrastructure of your organization (e.g., location, size, and groups that need tailored services) affect implementation of the new National Standards for Cancer Survivorship Care? | |
|  | **Culture**  Norms, values, and basic assumptions | 1. How do you think your organization’s culture (general beliefs, values, assumptions that people embrace) will affect the implementation of the National Standards for Cancer Survivorship Care? Can you please describe an example? | |
|  | **Implementation Climate**  The absorptive capacity for change, shared receptivity of involved individuals to an intervention and the extent to which use of that intervention will be rewarded, supported, and expected within their organization | ***Subconstruct:***  ***Tension for Change***  The degree to which stakeholders perceive the current situation as intolerable or needing change | 1. Do you believe there a strong need for the new National Standards for Cancer Survivorship Care? Why or why not? 2. How do you perceive that people feel about current standards and related programs/practices/processes that are available for survivorship care?    a. To what extent do you feel survivors’ care needs are already being met?    b. To what extent do you feel current programs/practices/processes fail to meet survivors’ needs? 3. What additional resources are needed? (Interviewer note: please refer to the slide deck, if needed) |
|  |  | ***Subconstruct: Relative Priority***  Individuals’ shared perception of the importance of the implementation within the organization | 1. To your knowledge, what kinds of high-priority survivorship care initiatives or activities are already happening in your setting? 2. To what extent might the implementation of these National Standards take a backseat or be consistent with other high-priority initiatives going on now, in your view? 3. What parts of the National Standards would you prioritize? (and why?) |
|  |  | ***Subconstruct: Goals & Feedback***  The degree to which goals are clearly communicated, acted upon, and fed back to staff and alignment of that feedback with goals | 1. Has your organization set goals related to survivorship care? If yes, how are these goals monitored for progress? |
|  |  | ***Subconstruct: Compatibility***  The degree of tangible fit between meaning and values attached to the intervention by involved individuals, how those align with individuals’ own norms, values, and perceived risks and needs, and how the intervention fits with existing workflows and systems | 1. How well do the National Standards for Cancer Survivorship Care fit within existing work processes and practices within your setting?    a. What are likely issues or complications that may arise? |
| Characteristics of Individuals | **Knowledge & Beliefs**  Individuals’ attitudes toward and value placed on the intervention as well as familiarity with facts, truths, and principles related to the intervention | 1. Do you think the National Standards for Cancer Survivorship Care would improve care if applied in your setting?    a. *Probe: How important is it to implement these standards to you?* 2. How do you feel about these new National Standards being applied in your setting? Do you have any feelings of anticipation, stress, enthusiasm? Why? | |
|  | **Self-efficacy**  Individual belief in their own capabilities to execute courses of action to achieve implementation goals | 1. How confident are you that you will be able to successfully implement the new National Standards for Cancer Survivorship Care in your practice? | |
| Process | **Engaging**  Attracting and involving appropriate individuals in the implementation and use of the intervention through a combined strategy of social marketing, education, role modeling, training, and other similar activities | **Key Stakeholders**   1. Who do you see as the key individuals to get on board with implementation of the National Standards for Survivorship Care? What are their roles? | |

Those are all of the questions planned for today. Is there anything else that you think would be helpful for me to know?

Thank you for your time. We may contact you again if we have any follow up questions.

Reference: <https://onlinelibrary.wiley.com/doi/full/10.1002/lrh2.10402>
